# Imaging of the Kidney and Urinary Tract: Current and Future Trends

**DOI:** 10.3390/medicina58050673

**Published:** 2022-05-19

**Authors:** Maria Assunta Cova, Fulvio Stacul, Michele Bertolotto

**Affiliations:** 1 Department of Radiology, University of Trieste, ASUGI, Ospedale di Cattinara, 34149 Trieste, Italy; bertolot@units.it; 2 Department of Radiology, University of Trieste, ASUGI, Ospedale Maggiore, 34125 Trieste, Italy; stacul.fulvio@gmail.com

The role of imaging in healthcare has become more and more significant in the last decades. Nowadays, imaging is crucial for diagnosis, decision making, treatment and follow-up in a large number of kidney and urinary tract disease. Radiologists are familiar with a number of techniques that offer diagnostic capabilities in these settings, such as Ultrasound (US), Computed Tomography (CT) and Magnetic Resonance Imaging (MRI). All such techniques recently faced major technical developments and now provide new diagnostic opportunities. Additionally, advanced imaging tools are enlarging our diagnostic capabilities. A major effort of continuous update is therefore required to radiologists in order to provide the best diagnostic opportunities to the patients. 

Furthermore, interventional radiology of the kidney and urinary tract is expanding. New fields have opened, and other fields can now be covered more in-depth thanks to technical advances.

This Special Issue of *Medicina* collects 10 papers dealing with a number of topics on imaging and interventional procedures and allows readers to focus on more recent advances and become familiar with their applications. In our opinion, it provides a useful and comprehensive update opportunity for radiologists and other physicians involved with kidney and urinary tract disease.

The first four papers deal with the diagnosis of renal masses. These papers underline the diagnostic capabilities of the different imaging modalities and show that integration of various modalities offers the greatest insight into characterization of renal masses. The detection of renal mass frequently occurs in the daily practice of Radiology Departments. US, contrast-enhanced ultrasound (CEUS), CT and MRI are commonly used to differentiate between benign and malignant lesions. Cystic lesions can be managed using the Bosniak classification. Management of solid lesions depends on whether the lesion is well defined or infiltrative. Radiologists may establish a possible differential diagnosis based on the imaging features of the renal masses and the clinical history. Diagnostic algorithms for the characterization of both well-defined and infiltrative solid renal masses can be suggested [1]. However, with the continuous advances in imaging diagnosis, new tools are being incorporated, such as CT texture analysis for the quantification of tumor heterogeneity, MRI diffusion and perfusion techniques, iodine quantification with dual-energy CT, and the use of lesion segmentation software to determine the degree of tumor enhancement. 

The role of CEUS is highlighted in the second and third papers of this Special Issue. CEUS can be used for assessing Bosniak III complex renal cystic lesions. It depicts a promising imaging modality for the precise diagnostic workup and stratification of renal cystic lesions, helping guidance of adequate clinical management [2]. CEUS can additionally be used for discriminating between benign and malignant solid renal masses (i.e., oncocytomas, angiomyolipomas and renal cell carcinomas). However, no specific sonomorphological characteristics allowing for accurate distinction between benign and malignant renal masses in CEUS could be detected [3].

The fourth paper focuses on the capabilities of CT to differentiate renal carcinomas. Quantitative multidetector CT (MDCT) enhancement patterns of renal masses can be useful to enable lesion differentiation by their enhancement characteristics. Such patterns can help distinguish clear cell renal cell carcinoma from malignant renal carcinoma subtypes and benign oncocytoma. These imaging features may contribute to providing prognostic information helpful in the management strategies of renal masses, avoiding a renal mass biopsy [4].

The following paper moves from diagnosis to treatment and highlights the capabilities of thermal ablation for minimally invasive treatment of renal carcinoma. Cryoablation is emerging as a safe and effective therapeutic option for treating renal cell carcinoma. Technical success, primary treatment efficacy and recurrence-free survival of the procedure have proven to be very close to surgical results in properly selected patients with very few major complications [5]. The multidisciplinary discussion plays fundamental role in choosing the best therapeutic approach for each patient with solid renal masses, patients with small renal tumors (pT1a) and patients at risk for surgery being the best candidates for the procedure.

The role of diagnostic imaging in patients with urinary tract infections is considered in the following paper. CT and MRI have become very important tools for the evaluation of urinary tract infections and for the detection of associated complications. Radiologists are expected to be confident with the radiological findings from CTs and MRIs in different types of upper and lower urinary tract infections, including acute pyelonephritis, renal abscesses, pyonephrosis, chronic pyelonephritis, fungal infections, cystitis, prostatitis and prostatic abscesses [6].

An additional two papers of this Special Issue highlight the capabilities of diagnostic imaging for evaluation of urothelial carcinomas. CT urography is now considered the imaging modality of choice for the diagnosis and staging of upper tract urothelial carcinoma. Some CT urography features help to differentiate low-risk tumors from high-risk tumors, leading to proper disease management. Additionally, the knowledge of possible upper tract urothelial carcinoma mimickers guides a correct differential diagnosis, avoiding mistreatment [7].

Nowadays conventional cystoscopy is still crucial in the diagnosis of bladder cancer, while staging can be performed with CT and MRI. However, a number of advanced imaging modalities can play a role in the diagnosis and follow-up of bladder tumors. They include advances in MRI (multiparametric MRI, where morphologic and functional imaging such as diffusion weighted imaging, diffusion tensor imaging and perfusion weighted imaging are used in combination), vesical imaging-reporting and data systems (VI-RADS), positron emission tomography (PET), the use of 18F-sodium fluoride PET/CT and bone scintigraphy, texture analysis and radiomics. However, further studies are needed to assess their full potential and correlation with the pathological findings [8].

Another paper focuses on vesicoureteral reflux, which is a common pediatric anomaly. In addition to the gold standard of voiding cystourethrography, contrast-enhanced voiding urosonography offers an excellent radiation-free alternative to voiding cystourethrography because of its high sensitivity. Therefore, it represents a good option in the diagnostic clarification of recurrent urinary tract infections with the suspected diagnosis of vesical ureteral reflux [9].

The last paper of this Special Issue deals with the increasing use of interventional radiology procedures of the male urogenital system. Such procedures provide effective and minimally invasive treatment options for multiple vascular, neoplastic, inflammatory and traumatic conditions of the urogenital system. At the kidney level, arterial embolizations are performed mainly for palliative treatment of parenchymal tumors, for renal traumas and, less frequently, for arteriovenous fistulas and renal aneurysms and pseudoaneurysms. Arterial embolization is also an effective therapy for intractable severe bladder hematuria secondary to a number of conditions, including unresectable bladder cancer. Endovascular interventional procedures for the penis are indicated for the treatment of post-traumatic priapism [10].

In conclusion, this Special Issue underlines the capabilities, advantages and limitations of various imaging modalities for the evaluation of renal and urinary tract disease. Additionally, it highlights opportunities offered by new imaging tools and gaps to be filled by future advances.
